# Wide-Field Optical Coherence Tomography in Patients with Diabetic Macular Edema

**DOI:** 10.3390/jcm13144242

**Published:** 2024-07-20

**Authors:** Krzysztof Kiciński, Maciej Gawęcki

**Affiliations:** 1 Department of Ophthalmology, Pomeranian Hospitals, 84-120 Wejherowo, Poland; krzysztofkg999@icloud.com; 2 Dobry Wzrok Ophthalmological Clinic, 80-822 Gdansk, Poland

**Keywords:** ultra-wide-field optical coherence tomography, choroidal thickness, retinal thickness, diabetic macular edema, diabetes

## Abstract

**Background**: The goal of the study was to analyze variations in central, perifoveal, and peripheral retinal thickness (RT) and choroidal thickness (CT) in patients with diabetic macular edema (DME) measured with ultra-wide-field optical coherence tomography (UWF-OCT). Additionally, correlations between RT and CT in the central, perifoveal, and peripheral sectors and the presence of selected systemic factors were evaluated. **Methods**: A total of 74 consecutive adult diabetic patients with DME and 75 healthy controls were included. Study participants were divided into three groups: DME patients without panretinal photocoagulation (PRP; 84 eyes), DME patients after PRP (56 eyes), and healthy controls (125 eyes). RT and CT were analyzed in three zones: a central circle of 3 mm diameter (central), a ring contained between a centered 9 mm circle and the central 3 mm circle (perifoveal), and a second, more peripheral ring between centered 18 mm and 9 mm circles (peripheral). Additionally, DME subgroups were analyzed according to the correlation of RT and CT with age, axial length, best corrected visual acuity (BCVA), diabetes duration, insulin therapy duration, body mass index (BMI), glycosylated hemoglobin (HbA1c) values, intravitreal injection (IVI) count, and the advancement of retinopathy assessed by the simplified diabetic retinopathy severity scale (DRSS). **Results**: The increase in RT in the far peripheral sectors in DME patients was not significant. The increases in central and perifoveal RT and lower values of CT in PRP-naive DME patients were strongly associated with poorer BCVA. Patients with DME after PRP presented with BCVA improvements significantly related to the number of IVIs. The amount of DME and RT in peripheral sectors were both independent of systemic factors such as BMI, duration of diabetes, duration of insulin intake, retinopathy severity, and HbA1c levels. **Conclusions**: Peripheral retinal sectors in DME patients are less affected in terms of increase in their thickness compared to central ones. Functional and morphological associations of DME with UWF-OCT testing refer to central and perifoveal sectors.

## 1. Introduction

The occurrence of diabetic macular edema (DME) has been associated with many systemic and local factors. The most important systemic associations include the duration of diabetes, glycemic control, insulin dependence, kidney functional status, and body mass index (BMI) [1,2,3,4]. Among local factors analyzed in the context of DME incidence are retinopathy severity, axial length, and choroidal thickness (CT) [5,6,7]. In modern ophthalmology, DME can be measured with software tools available in spectral domain optical coherence tomography (SD-OCT) devices. Such equipment enables the evaluation of retinal morphology and the measurement of central subfoveal thickness (CST), the parameter that, to some extent, characterizes DME severity. Modern OCT devices make it possible to measure CT as well. This anatomical structure plays an important role in nourishing the retina, and its impairment might result in retinal tissue alterations, DME notwithstanding. Associations between different factors and the amount of DME measured with SD-OCT have been analyzed in many studies engaging standard field devices [8,9,10,11]. Nevertheless, the employment of wide-field (WF) OCT in such analysis is rare, as this technology has just been introduced to clinical practice. WF-OCT with modern equipment provides CT and retinal thickness (RT) values in the peripheral sectors. It has been proved that evaluation of the retinal periphery plays an important role in determining the classification and risk of progression of diabetic retinopathy. The introduction of wide-field angiographic systems enabled more precise determination of the stage of diabetic retinopathy [12,13]. Moreover, the detection of peripheral lesions and areas of hypoperfusion by wide-field systems correlated with the risk of progression of retinopathy, as reported in some studies [14,15,16]. Increased retinal vascular bed area assessed with UWF fluorescein angiography was also associated with greater severity of DME [17], as well as poorer response to its intravitreal treatment [18]. That knowledge creates a possibility for employment of non-invasive wide-field diagnostic techniques, such as ultra-wide-field OCT (UWF-OCT) and ultra-wide-field angio-OCT (UWF-OCTA), for the purpose of evaluation of retinal and choroidal periphery in diabetic retinopathy. Besides non-invasive character, such techniques enable numerical analyses that provide additional solid information on the condition of the peripheral retina and choroid and its relationship to changes in the central sector. The goal of our study was to analyze variations in central, perifoveal, and peripheral RT and CT in patients with DME measured by ultra-wide-field OCT. Additionally, we sought correlations between RT and CT in the central, perifoveal, and peripheral sectors and the presence of selected systemic factors.

## 2. Materials and Methods

The study was conducted according to the Declaration of Helsinki and approved by the local ethical board of Dobry Wzrok Ophthalmological Clinic (No. 3/2024).

The study involved 74 consecutive adult patients treated for DME and diabetic retinopathy at the Specialist Hospital in Chojnice and 75 consecutive healthy adult controls in an occupational program examined in the outpatient clinic of the same hospital. All patients were subject to basic ophthalmological examinations that included anterior and posterior segment evaluation, intraocular pressure measurement, and best corrected visual acuity (BCVA) testing. Patients with opacity of optical media that prevented the acquisition of quality OCT scans were excluded from the study. Additionally, the DME patients’ medical histories were reviewed for diabetes duration, insulin dependence and duration of intake, current HbA1c (glycosylated hemoglobin) values, BMI, number of received intravitreal injections, and peripheral panretinal photocoagulation (PRP). As peripheral laser treatment might significantly affect RT and CT, patients with PRP were analyzed as a separate study subgroup. In all cases, PRP was performed longer than 6 months before the UWF-OCT measurements.

For the control group, eyes without ocular pathologies or previous ocular surgical treatments were initially qualified for WF-OCT testing. Patients with systemic diseases, such as diabetes, metabolic disorders, and hypercholesterolemia, or pregnancy, which could influence the results, were also excluded from the control group.

After the application of the exclusion criteria, the study participants were divided into three groups: DME patients without PRP (84 eyes), DME patients after PRP (56 eyes), and healthy controls (125 eyes). The demographics of the study and control groups are provided in Table 1. 

**Figure 1:**
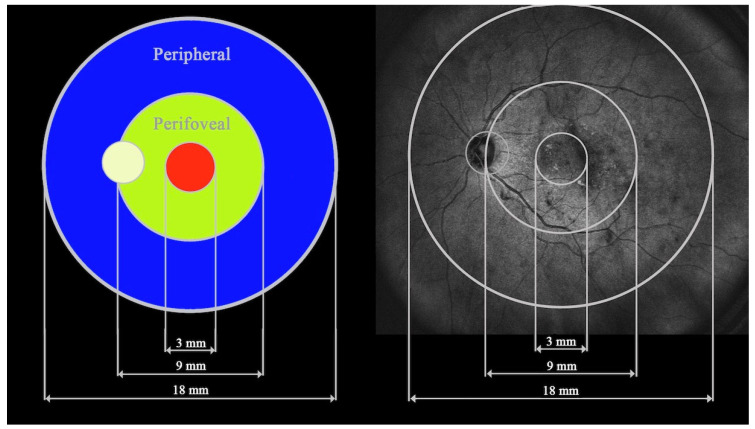
The three fields analyzed in the study: central, perifoveal, and peripheral.

All patients underwent WF-OCT testing performed after pupil dilation with swept-source Xephilio OCT-S1 (Canon Medical Systems Europe B.V., Amstelveen, The Netherlands, 2023), which enables the widest scan of 23 × 20 mm. Measurements are performed in 24 fields. For the purpose of this study, the 24 fields were merged into three zones for easier evaluation and differentiation between far and close periphery: a central circle of 3 mm diameter (central), a ring contained between a centered 9 mm circle and the central 3 mm circle (perifoveal), and a second, more peripheral ring between centered 18 mm and 9 mm circles (peripheral); see Figure 1.

The data obtained from the three groups were analyzed according to RT and CT in all three zones. Additionally, the DME subgroups (with and without PRP) were analyzed according to the correlation of RT and CT with age, axial length, BCVA, diabetes duration, insulin therapy duration, BMI, HbA1c values, intravitreal injection (IVI) count, and advancement of retinopathy assessed by the simplified diabetic retinopathy severity scale (DRSS; five stages) [19,20].

**Figure 2:**
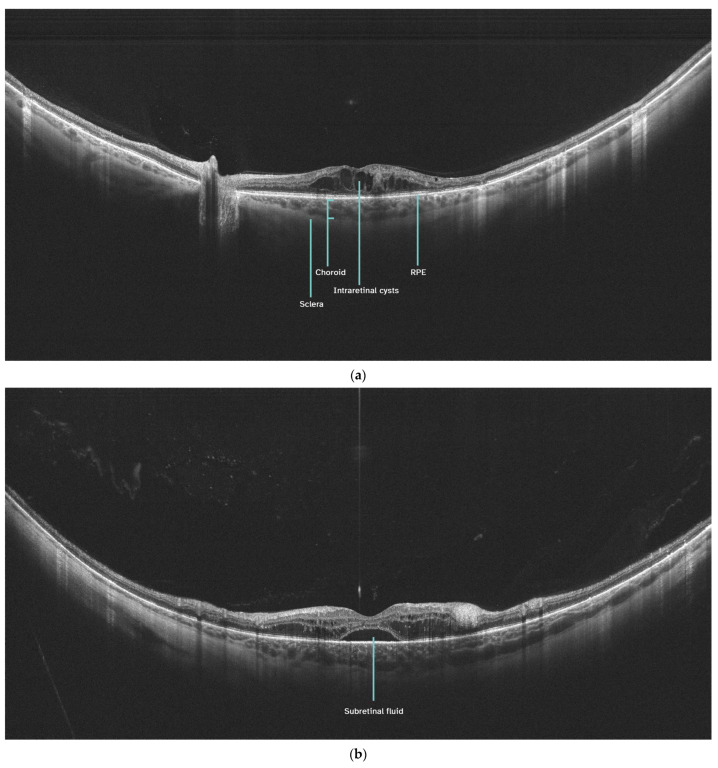
(**a**). UWF-OCT of significant DME with cystoid changes in the neurosensory retina. The scan engages the optic disc. (**b**). UWF-OCT of extensive DME with the presence of subretinal fluid. RPE—retinal pigment epithelium.

Examples of UWF-OCT scans of patients with DME are presented in Figure 2a,b.

### Statistical Procedures

Categorical variables were presented as integer numbers and percentages. Numerical traits were described by their mean, median, standard deviation, and lower-to-upper quartile values. The normality of the distribution was tested using the Shapiro–Wilk W test. Levene’s test was used to assess the homogeneity of variances. A multifactorial analysis of variance (ANOVA) without replications was performed to test the significance of differences in normally distributed numerical traits between the study groups. For non-normally distributed measures, generalized linear models were fitted. After the omnibus tests, post hoc multiple comparisons were carried out when applicable. A level of *p* < 0.05 was considered statistically significant. All tests and computations were performed using Statistica™, release 13.2 (TIBCO Software Inc., Palo Alto, CA, USA).

## 3. Results

All three groups were compared according to age, axial length, and received laser treatment. A significant variance of results was observed for BCVA, with the control group having significantly better BCVA but not for the other two analyzed factors. Table 2 presents the variance between the groups in age, axial length, BCVA, and number of received intravitreal injections. All patients were initially treated with intravitreal bevacizumab with a possible switch to aflibercept in unresponsive cases after a minimum of five injections.

The comparison of RT and CT between the three groups in the three defined sectors is presented in Table 3. No significant difference in CT was noted between the groups in any sector. Significant differences were observed in RT, however. The control group had the lowest RT values in all three sectors compared to the DME groups. The laser group presented with the highest RT values in all sectors; however, a statistically significant difference from the no laser group was observed for the perifoveal ring (*p* = 0.0125) but not the peripheral.

Table 4, Table 5, Table 6 and Table 7 present the relationships between analyzed factors and RT and CT for the DME subgroups. A significant relationship was found between BCVA and RT and for CT in the central and perifoveal sectors only for the subgroup not treated with laser. A lower CT correlated with older age in all sectors for the no laser group only. A higher IVI count strongly correlated with lower values of RT for the PRP subgroup of DME patients.

No other significant relationships were found for the study groups with reference to other analyzed factors: axial length, level of HbA1c, BMI, duration of diabetes, DRSS, and duration of insulin therapy.

## 4. Discussion

The subject of retinal and choroidal thickness in patients with diabetes mellitus, especially cases complicated by diabetic retinopathy, has been analyzed in many studies that employed standard field OCT [8,9,10,11]. WF-OCT testing has not been employed for such research so far, and our study is, to our knowledge, the first to do so. UWF-OCT provides additional information on peripheral RT and CT and thus enables evaluation of the involvement of this anatomical sector in diabetic retinopathy. As peripheral laser treatment (PRP) significantly affects RT and CT, correlations between DME and UWF-OCT measurements should be sought, particularly for laser-naïve cases. The results of the present study generally revealed similar patterns for RT and CT in the perifoveal and central sectors. An obvious increase in central RT in DME patients also extended to the perifoveal sectors. The retinal periphery, however, was affected to a lesser extent. DME patients who did not receive PRP had a peripheral RT that did not significantly differ from that of controls. Such a difference was, however, observed in patients who received PRP treatment and hence had severe non-proliferative or proliferative retinopathy. It is plausible that laser-induced peripheral retinal nerve fiber layer thinning was compensated in these patients, but a tendency for retinal thickening due to retinopathy was present [21,22].

Significant variations in CT in DME patients versus controls were not noted in our work in any sector. This outcome was also reported by other authors, who did not link the central RT with central CT in DME patients [23]. On the other hand, some studies showed long-term reductions in subfoveal CT after PRP [24,25]. It must be noted, however, that our study involved the analysis of CT in larger sectors, and it is plausible that mean CT variations after PRP in larger areas are smaller compared to those observed and reported in the limited area under the fovea.

The morphological findings of the UWF-OCT measurements in DME patients were also reflected in retinal function. PRP-naïve patients presented with BCVA strongly correlated with RT in the central and perifoveal sectors: better BCVA was related to a lower RT in these regions. The peripheral retina, however, did not show such a relationship. On the other hand, patients who received laser treatment did not present with such a correlation at all. Previous research showed that retinal architecture, not only RT, affects visual outcomes in diabetic retinopathy (DR) [26]. Moreover, in a milestone Diabetic Retinopathy Clinical Research network (DRCR net) group study, only a moderate correlation between central point RT and BCVA was noted [27]. In our research, a significant correlation between RT and BCVA in laser treatment-naive patients was found for larger central areas (3 mm and 9 mm in diameter). Hence, it can be speculated that the correspondence of RT and BCVA in DR is noted at its earlier stages and in cases with an extension of retinal thickening to parafoveal regions. Possibly, mean values of RT in larger sectors might serve as a predictor of preservation of visual function in DR.

Smaller CT values at the retinal center and perifoveal sector correlated with worse BCVA in laser-naïve patients. Again, such a relationship was not noted for the choroidal periphery. Hence, the condition of the peripheral choroid does not affect visual function as much as its center. The involvement of CT in visual outcomes in DR was analyzed in other standard field studies. A higher CT was a predictor for better BCVA improvements after anti-VEGF therapy [28]. A lower central CT was also associated with more severe retinopathy that presented with poorer BCVA [29]. These findings remain in consent with our results, proving better BCVA in cases with greater central CT.

CT in DME patients has a strong correlation to the patient’s age. This relationship, assessed in patients who did not receive laser treatment, proved to be true for all sectors, including the choroidal periphery. Patients after PRP showed such a relationship only for the central choroidal sector. A decrease in CT with age in all sectors was observed in healthy individuals as well, as shown by a previous UWF-OCT publication by the same authors [30]. Thus, it can be stated that the sole presence of DME does not influence this correlation in a significant way.

The number of received IVIs was significantly related to a reduction in RT in all sectors, but only for patients after PRP. Such a relationship was not noted for patients who received IVI treatment alone. Thus, it can be speculated that peripheral retinal ablation in DR provides more consistent morphological outcomes of IVI management with reference to the retinal center. Intravitreal therapy without peripheral laser in DR was associated with a higher variance of RT despite the received treatment. Nevertheless, recent high-quality research did not show a reduction in the number of IVIs or differences in BCVA improvement in patients with DME who underwent PRP targeted at ischemic areas [31,32]. Hence, a more straightforward reaction to IVI treatment does not necessarily mean a lower number of required injections in the long term.

The lack of correlation between RT and CT and other factors, especially systemic ones, proves that morphological changes of the choroid and retina in diabetic retinopathy are related to multiple factors, not just selected ones. Interestingly, RT and CT were independent of DRSS grading. This finding proves that RT in DME patients does not necessarily increase consent with the severity of retinopathy. It is difficult to relate our results to other published research. Only a few studies analyzed the relationship between DME severity and systemic factors in diabetes. These studies proved such relationships only for systemic inflammatory biomarkers [33,34,35]. Other systemic factors were analyzed in the context of DME incidence or response to its treatment, not specific RT and CT values [36,37,38,39]. Among such factors, high levels of HbA1c or a lower estimated glomerular filtration rate were named most often.

Analysis of UWF-OCT testing in DME shows lesser involvement of far peripheral sectors compared to central and perifoveal ones. Nevertheless, UWF-OCT devices provide solid and easily accessible information on RT and CT in the posterior pole ring (located between central 3 and 9 mm circles), which is significantly affected in diabetic retinopathy complicated by the DME. We believe that it is sensible to include that area in further analysis of factors influencing DME incidence, severity, and response to treatment.

## 5. Conclusions

UWF-OCT provides information on peripheral retinal and choroidal involvement in diabetic retinopathy complicated by DME. Peripheral retinal sectors in DME patients are less affected in terms of increase in their thickness compared to central ones. An increase in central and perifoveal RT in PRP-naive patients with DME is strongly associated with poorer BCVA, while higher central and perifoveal CT values refer to better visual acuity in these patients. An association of peripheral RT and CT with BCVA was not found. Patients with DME after PRP present with BCVA improvements significantly related to the number of IVIs. The amount of DME and RT in peripheral sectors was independent of systemic factors such as BMI, duration of diabetes, duration of insulin intake, and HbA1c levels.

## Figures and Tables

**Table 1:** Baseline characteristics of the study cohort by prevalence of diabetes mellitus and laser application (discrete variables).

Analyzed Trait	Study Group			*p* Value
	Control Group	DME w/Laser	DME w/o Laser	
No. of participants, n (%)	75 (50.33)	32 (21.48)	42 (28.19)	
No. of eyes, n (%)	125 (47.17)	56 (21.13)	84 (31.70)	
Gender, n (%)				
- Female	44 (58.67)	12 (37.50)	18 (42.86)	=0.0779
- Male	31 (41.33)	20 (62.50)	24 (57.14)	

w—with, w/o—without, DME—diabetic macular edema.

**Table 2:** Baseline characteristics of the study cohort by prevalence of diabetes mellitus and laser application (numerical variables); n = 149 individuals = 265 eyes.

Analyzed Trait	DME/Laser	Statistical Parameter *				*p* Value **
		Mean	SD	Median	Q_1_–Q_3_	
Age [years]	DME w/laser	60.22	13.03	66.00	51.00–69.00	=0.1448
	DME w/o laser	63.67	9.17	64.00	57.50–71.50	
	Control group	59.03	15.92	63.00	48.00–71.00	
Axial length [mm]	DME w/laser	24.76	1.47	24.40	23.75–26.10	=0.3145
	DME w/o laser	25.03	1.54	25.00	24.10–25.80	
	Control group	24.78	1.59	24.80	23.80–25.60	
BCVA (logMAR)	DME w/laser	0.56	0.36	0.55	0.30–0.70	0.0008
	DME w/o laser	0.32	0.28	0.30	0.10–0.50	
	Control group	0.00	0.00	0.00	0.00–0.00	
Minimal IVI count	DME w/laser	6.67	3.19	5.00	4.00–10.00	0.6119
	DME w/o laser	6.71	2.73	5.00	5.00–9.00	
	Control group	0.00	0.00	0.00	0.00–0.00	

(* Statistical measures used: SD—standard deviation, Q—quartile. ** Controlled for gender and/or age.) DME—diabetic macular edema, BCVA—best corrected visual acuity, w—with, w/o—without, IVI—intravitreal injections.

**Table 3:** Descriptive statistics for the retinal/choroidal thicknesses (µm) of the study cohort by prevalence of diabetes mellitus and laser application (n = 265 eyes).

Measured Field	DME/Laser	Statistical Parameter				*p* Value *
		Mean	SD	Median	Q_1_–Q_3_	
Retinal thickness						
Central	DME w/laser	369.79	66.84	363.00	313.00–394.00	<0.0001 ^a^
	DME w/o laser	366.36	61.42	351.00	325.00–380.50	
	Control group	335.08	20.69	335.00	324.00–348.00	
Perifoveal	DME w/laser	315.28	39.77	307.78	281.41–337.91	<0.0001 ^b^
	DME w/o laser	302.20	30.00	296.47	283.72–313.72	
	Control group	287.50	14.98	289.81	277.37–297.75	
Peripheral	DME w/laser	227.07	21.06	219.19	212.37–341.63	=0.0150 ^c^
	DME w/o laser	225.60	17.32	220.94	214.62–234.94	
	Control group	220.29	12.43	221.37	211.50–228.63	
Choroidal thickness						
Central	DME w/laser	288.18	76.63	293.00	220.50–353.50	=0.9227
	DME w/o laser	285.82	85.33	288.00	230.50–352.00	
	Control group	293.87	87.10	300.00	223.00–357.00	
Perifoveal	DME w/laser	254.89	75.90	240.72	201.56–309.31	=0.7573
	DME w/o laser	244.75	68.90	231.47	193.53–284.31	
	Control group	250.98	71.48	241.00	200.69–314.19	
Peripheral	DME w/laser	196.54	56.46	183.56	159.00–227.19	=0.2139
	DME w/o laser	185.10	45.71	176.06	153.06–206.63	
	Control group	186.57	46.02	174.12	152.62–221.00	

(* Controlled for the study subjects’ age and gender. Results of post hoc comparisons: ^a^. DME w/laser vs. control group *p* = 0.0004, DME w/laser vs. DMW w/o laser *p* = 0.8772, DME w/o laser vs. control group *p* = 0.0001; ^b^. DME w/laser v. DME w/o laser *p* = 0.0126, DME w/laser vs. control group *p* = 0.0011, DME w/o laser vs. control group *p* < 0.0001; ^c^. DME w/laser vs. control group *p* = 0.0457, DME w/laser vs. DME w/o laser *p* = 0.7867, DME w/o laser vs. control group *p* = 0.0812.) DME—diabetic macular edema, SD—standard deviation, w—with, w/o—without, Q—quartile.

**Table 4:** Pearson’s product moment correlation coefficients along with corresponding *p* values for the retinal/choroidal thicknesses versus selected continuous traits in the DM group without the use of laser.

Patient Characteristics	Axial Length (mm)		BCVA (logMAR)		HbA1c (%)		BMI (kg/m^–2^)		Age (Years)	
Measured Field	*r*	*p*	*r*	*p*	*r*	*p*	*r*	*p*	*r*	*p*
Retinal thickness										
Central	–0.11	0.3385	0.33	0.0025	0.001	0.9904	–0.20	0.2320	0.11	0.3086
Perifoveal	0.06	0.5659	0.23	0.0384	0.08	0.5240	–0.15	0.3818	–0.07	0.5473
Peripheral	0.30	0.0052	0.16	0.1411	–0.03	0.7980	–0.10	0.5468	–0.14	0.1916
Choroidal thickness										
Central	0.20	0.0656	–0.25	0.0223	–0.04	0.7082	–0.12	0.4593	–0.44	<0.0001
Perifoveal	0.21	0.0518	–0.26	0.0187	–0.07	0.5467	–0.11	0.5322	–0.48	<0.0001
Peripheral	0.18	0.1084	–0.15	0.1796	–0.14	0.2306	0.02	0.8998	–0.38	0.0004

BCVA—best corrected visual acuity, BMI—body mass index, HbA1c—glycosylated hemoglobin.

**Table 5:** Spearman’s rank correlation coefficients along with corresponding *p* values for the retinal/choroidal thicknesses versus selected integer or non-normally distributed traits in the DM group without the use of laser.

Patient Characteristics	DM Duration (Years)		Insulin Therapy (Years)		IVI Count		DRSS	
Measured Field	*r*	*p*	*r*	*p*	*r*	*p*	*r*	*p*
Retinal thickness								
Central	–0.11	0.3707	–0.22	0.1256	–0.15	0.3103	–0.13	0.3009
Perifoveal	–0.19	0.1220	–0.15	0.3078	–0.17	0.2580	0.19	0.1213
Peripheral	0.01	0.9322	0.20	0.1700	0.10	0.5131	0.07	0.5730
Choroidal thickness								
Central	–0.14	0.2557	0.28	0.0531	0.13	0.4047	–0.06	0.6371
Perifoveal	–0.15	0.2313	0.25	0.0910	0.06	0.6952	–0.08	0.5064
Peripheral	–0.16	0.1828	0.15	0.2982	0.07	0.6251	–0.10	0.4545

DM—diabetes mellitus, IVI—intravitreal injection, DRSS—diabetic retinopathy severity scale.

**Table 6:** Pearson’s product moment correlation coefficients along with corresponding *p* values for the retinal/choroidal thicknesses versus selected continuous traits in the DM group after laser treatment.

Patient Characteristics	Axial Length (mm)		BCVA (logMAR)		HbA1c (%)		BMI (kg/m^–2^)		Age (Years)	
Measured Field	*r*	*p*	*r*	*p*	*r*	*p*	*r*	*p*	*r*	*p*
Retinal thickness										
Central	–0.02	0.9030	0.01	0.9362	0.06	0.6852	–0.08	0.7058	0.13	0.3548
Perifoveal	0.10	0.4693	0.13	0.3667	0.08	0.5709	–0.15	0.5069	0.04	0.7504
Peripheral	0.36	0.0066	0.24	0.0870	–0.11	0.4549	–0.06	0.7869	–0.13	0.3627
Choroidal thickness										
Central	–0.16	0.2495	–0.05	0.7246	0.08	0.5955	0.27	0.2213	–0.31	0.0213
Perifoveal	–0.14	0.3171	–0.07	0.6144	0.15	0.3035	0.14	0.5195	–0.25	0.0693
Peripheral	–0.08	0.5485	–0.01	0.9365	–0.01	0.9667	–0.03	0.9001	–0.18	0.1859

BCVA—best corrected visual acuity, BMI—body mass index, HbA1c—glycosylated hemoglobin.

**Table 7:** Spearman’s rank correlation coefficients along with corresponding *p* values for the retinal/choroidal thicknesses versus selected integer or non-normally distributed traits in the DM group after laser treatment.

Patient Characteristics	DM Duration (Years)		Insulin Therapy (Years)		IVI Count		DRSS	
Measured Field	*r*	*p*	*r*	*p*	*r*	*p*	*r*	*p*
Retinal thickness								
Central	–0.27	0.0837	–0.30	0.0606	–0.38	0.0504	0.02	0.8886
Perifoveal	–0.28	0.0656	–0.38	0.0171	–0.61	0.0006	0.17	0.3036
Peripheral	–0.09	0.5611	–0.10	0.5364	–0.49	0.0088	0.31	0.0519
Choroidal thickness								
Central	–0.24	0.1279	0.16	0.3326	–0.001	0.9963	0.05	0.7673
Perifoveal	–0.16	0.3008	0.26	0.1144	0.04	0.8574	0.04	0.8128
Peripheral	–0.02	0.8748	0.29	0.0761	–0.10	0.6279	–0.002	0.9883

DM—diabetes mellitus, IVI—intravitreal injections, DRSS—diabetic retinopathy severity scale.

## Data Availability

Supporting data are available upon reasonable request.
